# Molecular epidemiology of human enterovirus 71 at the origin of an epidemic of fatal hand, foot and mouth disease cases in Cambodia

**DOI:** 10.1038/emi.2016.101

**Published:** 2016-09-21

**Authors:** Veasna Duong, Channa Mey, Marc Eloit, Huachen Zhu, Lucie Danet, Zhong Huang, Gang Zou, Arnaud Tarantola, Justine Cheval, Philippe Perot, Denis Laurent, Beat Richner, Santy Ky, Sothy Heng, Sok Touch, Ly Sovann, Rogier van Doorn, Thanh Tan Tran, Jeremy J Farrar, David E Wentworth, Suman R Das, Timothy B Stockwell, Jean-Claude Manuguerra, Francis Delpeyroux, Yi Guan, Ralf Altmeyer, Philippe Buchy

**Affiliations:** 1 Pasteur Institute in Cambodia, Phnom Penh 12000, Cambodia; 2 Pasteur Institute, Paris 75724, France; 3 State Key Laboratory of Emerging Infectious Diseases, School of Public Health, The University of Hong Kong, Hong Kong, China; 4 Institut Pasteur in Shanghai, Shanghai 200025, China; 5 PathoQuest, Paris 75015, France; 6 Kantha Bopha Hospital, Phnom Penh 12000, Cambodia; 7 Ministry of Health, Phnom Penh 12000, Cambodia; 8 Oxford University Clinical Research Unit, Hospital for Tropical Diseases, Ho Chi Minh P1Q5, Vietnam; 9 J. Craig Venter Institute, Rockville, MD 92037, USA; 10 National Institute for Health and Medical Research, INSERM U994, Paris 75000, France; 11 GlaxoSmithKline Vaccines R&D, Singapore 189720, Singapore

**Keywords:** Cambodia, epidemic, hand foot and mouth disease, human enterovirus 71, severe disease

## Abstract

Human enterovirus 71 (EV-A71) causes hand, foot and mouth disease (HFMD). EV-A71 circulates in many countries and has caused large epidemics, especially in the Asia-Pacific region, since 1997. In April 2012, an undiagnosed fatal disease with neurological involvement and respiratory distress occurred in young children admitted to the Kantha Bopha Children's Hospital in Phnom Penh, Cambodia. Most died within a day of hospital admission, causing public panic and international concern. In this study, we describe the enterovirus (EV) genotypes that were isolated during the outbreak in 2012 and the following year. From June 2012 to November 2013, 312 specimens were collected from hospitalized and ambulatory patients and tested by generic EV and specific EV-A71 reverse transcription PCR. EV-A71 was detected in 208 clinical specimens while other EVs were found in 32 patients. The VP1 gene and/or the complete genome were generated. Our phylogenetic sequencing analysis demonstrated that 80 EV-A71 strains belonged to the C4a subgenotype and 3 EV-A71 strains belonged to the B5 genotype. Furthermore, some lineages of EV-A71 were found to have appeared in Cambodia following separate introductions from neighboring countries. Nineteen EV A (CV-A6 and CV-A16), 9 EV B (EV-B83, CV-B3, CV-B2, CV-A9, E-31, E-2 and EV-B80) and 4 EV C (EV-C116, EV-C96, CV-A20 and Vaccine-related PV-3) strains were also detected. We found no molecular markers of disease severity. We report here that EV-A71 genotype C4 was the main etiological agent of a large outbreak of HFMD and particularly of severe forms associated with central nervous system infections. The role played by other EVs in the epidemic could not be clearly established.

## INTRODUCTION

Enteroviruses (EVs) belong to the genus *Enterovirus* within the family *Picornaviridae*, which are small non-enveloped, single-stranded positive-sense RNA viruses with a genome of 7500 bases.^1^ Human enteroviruses (HEV) are classified into three main species: EV-A, EV-B, EV-C, and other less common EV D-J species.^1, 2^ Human enterovirus 71 (EV-A71) belongs to the EV-A species, which along with other EVs can cause hand, foot and mouth disease (HFMD).^3^

EV-A71 was first isolated in California in 1969,^4^ although it was suggested that the virus may have circulated in the Netherlands as early as 1963.^5^ EV-A71 is present in most countries. Large-scale epidemics, however, have been observed in Asia-Pacific since 1997. EV-A71 has become endemic in this region, with regular emergences of new genetic lineages.^6^

Sequencing of the P1 coding region for capsid proteins is well correlated with the previous definition of serotype. The highly variable sequences of the EV-A71 VP1 gene have been used to group EV-A71 isolates into four genotypes (A, B, C and D).^7, 8^ EV strains of the same genotype have >92% nucleotide sequence identity, whereas isolates with a diversity of >15% are considered as different genotypes.^9^ The A group consists of viruses isolated in the United States of America in 1969, including the prototype BrCr strain.^4^ Genotype B has five subgenotypes and has been circulating in Asia, particularly in Malaysia and Singapore, since the 1970s.^10^ Subgenotype B5 was reported during recent outbreaks in Thailand,^11, 12^ Taiwan^13, 14^ and Vietnam.^15^ Genotype C also comprises five subgenotypes. C1 emerged in the mid-1980s^10^ and replaced genotype B as the predominant genotype in Europe and America.^10^ Subgenotype C2 emerged in 1995 in Australia and the United States of America.^10^ The C3 subgenotype emerged in Korea and Japan.^16, 17^ C4 has circulated predominantly in China since 2000 and is present in many other Asian countries.^18, 19, 20, 21^ The C5 subgenotype was reported in Japan, Vietnam and Taiwan.^20, 22^ Other less frequent EV-A71 genotypes (D–J) have been isolated in India, Africa and Madagascar.^23^

In recent years, numerous HFMD outbreaks were reported in Asian countries, including China, Vietnam, Singapore, South Korea and Thailand.^24^ In April 2012, an undiagnosed fatal disease with neurological involvement and respiratory failure was detected at Kantha Bopha Children's Hospital in Phnom Penh, Cambodia. Most of the children who were affected were aged below three years and mainly originated from the southern and central parts of the country.^25^ Most of them died (56/61 cases), usually within a day after admission to the hospital.^25^ Etiological and epidemiological investigations were initiated in June 2012 after the first series of clinical samples were submitted to the Virology Unit at Institut Pasteur in Cambodia (IPC), Phnom Penh, Cambodia. EV-A71 followed by other non-EV-A71 EVs were the microorganisms that were most commonly detected in these patients.^26^ The present study aims to describe the EV genotypes that were isolated during the outbreak in 2012 and 2013.

## MATERIALS AND METHODS

### Sample collection

From June 2012 to November 2013, clinical specimens such as throat, nasopharyngeal and rectal swabs, cerebrospinal fluid (CSF), bronchoalveolar lavage and sera were collected from hospitalized patients and outpatients at Kantha Bopha Hospital in Phnom Penh and Jayavarman VII Hospital in Siem Reap (Northwest Cambodia).

Patients who were admitted to the two hospitals with signs and symptoms of EV infection (i.e., all patients hospitalized with severe disease as well as some patients randomly selected in the outpatient ward presenting with mild disease) were classified into the following three main clinical categories: HFMD, HFMD with central nervous system involvement (CNSI) alone and HFMD with cardiopulmonary failure (CPF). All patients in the CPF group also experienced CNSI.^27^

The patient demographic data, clinical symptoms, diagnostic test results and medical imaging findings were collected by the clinical team. Because the initial series of samples used in this study were collected for diagnostic purposes and characterization of the microorganism involved in an outbreak setting, approval was not sought from the National Ethics Committee. Once the causative agent was identified, patients with encephalitis were included in an ongoing study on the etiology of pediatric encephalitis; this study was approved by the National Ethics Committee (approval no. 107NECHR). Additional authorization was obtained to include the outpatients presenting with HFMD (approval no. 0059NECHR). For all patients, a signed informed consent was obtained from their guardians before inclusion.

### EV-A71 diagnosis

#### Diagnostic reverse transcription PCR (RT-PCR)

RNA was extracted from 140 μL of rectal, throat and nasopharyngeal swabs collected in viral transport medium, serum and CSF using the QIAamp Viral RNA Mini Kit (Qiagen1, Hilden, Germany) according to the manufacturer's recommendations.

A specific real-time RT-PCR assay was used for the detection of EV-A71 as described elsewhere.^28^ Briefly, five μL of RNA was reverse-transcribed and amplified using the SuperScript III Platinum One-Step qRT-PCR Kit (Invitrogen, Carlsbad, CA, USA) in a mixture of 10 μL of 2 × Reaction Mix with ROX, 10 μM forward primer (EV71-VP1-634F: 5′-GGA GAA CAC AAR CAR GAG AAA GA-3′), 10 μM reverse primer (EV71-VP1-743R: 5′-ACT AAA GGG TAC TTG GAC TTV GA-3′), 10 μM of EV-A71-specific probe (EV71-VP1-TaqMan: 5′-FAM-TGA TGG GCA CGT TCT CAG TGC G-BHQ1-3′) and one μL of SuperScript III RT/Platinum Taq Mix. Molecular-grade water was added to the reaction to obtain a final volume of 20 μL. Thermocycling was conducted as follows: reverse transcription at 50 °C for 30 min, then holding at 95 °C for 2 min, followed by 40 cycles of 95 °C for 15 s, 55 °C for 30 s and 72 °C for 20 s.

The samples that tested negative for EV-A71 were further investigated using pan-EV real-time RT-PCR adapted from Beld *et al.*,^29^ using a few modifications for the detection of other HEVs. Specifically, the RT step was performed prior to TaqMan real-time PCR. The extracted RNAs were reverse-transcribed using random hexamers. The RT was conducted by adding seven μL of RNA into a mixture containing 1500 ng of random hexamers and 10 mM of each deoxynucleoside triphosphate. Molecular-grade water was added to obtain the final volume of 12 μL. After incubation for five min at 60 °C and one min on ice, seven μL of the second mix (containing 4 μL of 5 × buffers, 0.1 M of DTT, 40 U of RNaseOut and five U of SuperScript III Reverse Transcriptase (Invitrogen) and 1.4 μL of molecular-grade water) was added to the first mixture. The second mixture was incubated at 25 °C for 10 min, at 50 °C for one h and at 72 °C for 15 min.

#### Virus isolation

Patient's samples were inoculated onto Vero E6 cells. Briefly, 150 μL of CSF, 20 μL of sera or 100 μL of rectal, nasopharyngeal and throat swab samples collected in viral transport medium were added to 80% confluent Vero E6 cells in a 12-well plate. After incubating for 1 h at 37 °C with 5% CO_2_, 1.7 mL of maintenance medium (Dulbecco's Modified Eagle Medium+2% of heat inactivated fetal calf serum and 1% of Penicillin–Streptomycin (Sigma-Aldrich, Saint Louis, MO, USA)) was added to each well. The plate was then incubated for seven days with the same incubation conditions. The presence of the virus in the supernatant was revealed by the diagnostic real-time PCR method described above.

### Sequencing of EV-A71

#### Sequencing of the VP1 gene

RNA from the PCR-positive cell culture supernatants was extracted using the QIAamp Viral RNA Mini Kt (Qiagen, Hilden, Germany) and the VP1 gene was sequenced using the primers described by Nix *et al.*^30^ DNA sequencing was performed in a commercial facility using an ABI 3730XL Analyzer (96-capillary) using the ABI Prism BigDye Terminator Cycle Sequencing Kit according to the manufacturer's instructions (Macrogen, Seoul, South Korea).

#### Whole-genome sequencing

Whole-genome sequencing of the high-titrated cell culture supernatants was performed using high-throughput sequencing. The culture supernatants of two isolates were clarified by centrifugation at 3500–4000 r for 10 min at 4 °C to remove the cell debris. They were then ultracentrifuged at 50 000 r.p.m. to collect the viral pellet. The pellet was resuspended in 140 μL of 10 mM Tris-Cl buffer (pH 7.5) and treated with RNase, followed by the addition of 560 μL of prepared AVL Buffer (Qiagen) not containing carrier RNAs to cleave the viral particles. RNA extraction was conducted using the QIAamp Viral RNA Mini Kit (Qiagen) and DNAs were removed using the RNase-Free DNase Set (Qiagen). Double-stranded cDNAs were synthesized using the cDNA Synthesis System (Roche Applied Science, Penzberg, Germany) and random amplification of the viral cDNAs was then carried out as previously described.^31^ Whole-genomic sequencing was conducted on a Genome Sequencer Junior (Roche Applied Science), and the original reads were assembled into contigs using Lasergene, version 9.0 (http://www.dnastar.com).

For sample preparation, sequencing and bioinformatic analyses were conducted as described in Gagnieur *et al.*,^32^ except that the human reads were filtered against NCBI build 37.1/assembly hg19. Each sample was sequenced with a depth ranging from 67 to 233 million reads each.

#### Whole-genome sequencing by Sanger method

The total RNA was extracted from biological samples or cell culture suspension using the QIAamp Viral RNA Mini Kit (Qiagen) as described above. cDNA was synthesized from 3 μL of RNA using two μL of each nucleotide triphosphate (Eurobio, Mannheim, Germany), 10 pmol of each EV-A71 primer (Supplementary Table S1) and 7.5 μL of sterilized distilled water. The mixture was incubated at 70 °C for 5 min and then stored on ice for 2 min. Each tube was then incubated with 1 μL of SuperScript II Reverse Transcriptase (Invitrogen, Life Technologies) and 0.5 μL of RNasin (Promega, Madison, WI, USA) in a final volume of 20 μL for 50 min at 42 °C, then at 95 °C for 5 min and at 4 °C for 1 min.

5 μL of cDNA was then amplified by sequencing PCR using 10 pmol of paired primers for each EV-A71 fragment (Supplementary Table S1), one μL of AmpliTaq DNA polymerase (Applied Biosystems, Carlsbad, CA, USA; Life Technologies), 10 nmol of each nucleotide triphosphate and 10 × taq buffer for a total of 5 μL, in a final volume of 50 μL with H_2_O added as necessary. The cycling conditions included initial denaturation at 94 °C for 3 min, 35 cycles of denaturation at 94 °C for 30 s, annealation at 55 °C for 30 s, extension at 72 °C for 1 min and final extension at 72 °C for 5 min. The PCR amplicons were sequenced by the Sanger method at a commercial facility (Macrogen).

All partial VP1 and full-genome sequences generated in this study were submitted to the GenBank sequence database (NCBI, Bethesda, MD, USA) with accession numbers as follows: KX197414–KX197422 (EV-A71 VP1), KX197423–KX197454 (Non EV-A71, VP1) and KX197455–KX197465 (EV-A71 full genome) (Supplementary Table S2).

### Sequence analysis and phylogenetic analysis

Sequences generated from the PCR products obtained for each strain were analyzed and assembled using the CLC Main Workbench 5.5 package (CLC bio A/S, Aarhus, Denmark). The EV-A71 reference strains consist of 242 sequences of the VP1 gene and 457 full-genome sequences available in GenBank belonging to two genotypes: B1–B5 and C1–C5. For the phylogenetic analysis of the other EV isolates, 108 VP1 sequences of reference strains, including all four EV species (A, B, C and D), were used.

A Parechovirus (JX575746) sequence was used as an out-group in the phylogenetic analysis. Multiple sequence alignment of Cambodian strains with reference strains available in GenBank was conducted using Muscle,^33^ which is available in the Seaview version 4.2.5 package.^34^ Phylogenetic analyses were performed using the maximum likelihood method using the GTR model suggested by Jmodeltest,^35^ which is available in MEGA 5.2^36^ with 1000 bootstrap re-sampling.

### Recombination analysis

We used version 3 of the Recombinant Detection Program (RDP 4.56)^37^ to screen for recombination in our set of EV-A71 full-genome sequence data. This program implements several methods for the identification of recombinant sequences and recombination breakpoints. Accordingly, we used the RDP,^38^ LARD,^39^ Bootscanning,^40^ Maxchi,^41^ Chimaera,^42^ GeneConv,^43^ 3Seq^44^ and Sis-scan^45^ methods, using the default settings in each case. We only considered recombination events that were identified by at least three methods. Statistical significance was set to the *P*<0.01 level after considering the Bonferroni correction for multiple comparisons as implemented in RDP.

### Statistical analyses

The means of continuous variables and the proportions of the categorical variables were compared using Student's *t*-test, Chi^2^ test and Fisher's exact test as appropriate. Statistical significance was set to 0.05. All statistical analyses were carried out using the Stata 12 statistical software for Windows (StataCorp LP, College Station, TX, USA).

## RESULTS

### Patient characteristics and EV genotyping

Between June 2012 and November 2013, 142 cases of HFMD, 20 cases with CNSI and 150 cases of encephalitis with CPF were recruited by the two participating hospitals for inclusion in the present study (Table 1). Among the suspected cases, EV-A71 was detected by either RT-PCR and/or virus isolation in 96 (67.6%) of the HFMD patients, 11 (55.0%) patients with CNSI and 101 (67.3%) patients with CNSI associated with CPF. Additionally, co-infection with EV-A71 and non-EV-A71 was detected in five cases. This was immediately reported to the health authorities. The differences in EV-A71 prevalence by disease severity group were not statistically significant (*P*=0.389).

The children included in this study were relatively young in all three clinical groups, with mean ages of 2.18 (95% confidence interval (CI): 1.78–2.58), 2.51 (95% CI: 1.04–4.03) and 2.84 (95% CI: 2.28–3.40) years in HFMD, CNSI and CPF cases, respectively (Table 1). There was no significant association between the age of the children and the severity of disease (*P*=0.92). However, there were significantly more girls in CPF cases than in HFMD (*P*=0.023) except in ‘pure' encephalitic syndromes (*P*=0.33).

The case fatality rate (CFR) was higher among CPF compared with CNSI cases (63.3% vs 15.0%, *P*<0.001). No fatalities were reported in HFMD patients.

Laboratory-confirmed EV-A71 and other EV cases originated from 22 of the 24 Cambodian provinces. Most cases, however, came from the northwestern (78/240) and southern (98/240) parts of the country. This pattern corresponds both to the density of the population and to the catchment area of the two participating hospitals (Supplementary Figure S1).

Non-EV-A71 viruses were detected in 37 cases (11.9%). Among these, five were co-infections with EV-A71. Additionally, five EVs detected by generic RT-PCR were unsubtypeable (VP1 sequencing attempt failed) (Table 1). Of all the non-EV-A71 viruses, EV-A was the most frequent (59.5%) followed by EV-B (28.0%) and EV-C (12.5%). The mean age of non-EV-A71 cases was two years (*n*=36; 95% CI: 1.54–2.77; range 0.25–7). The sex ratio was 0.76 males per female.

### Phylogenetic analysis

**Figure 1:**
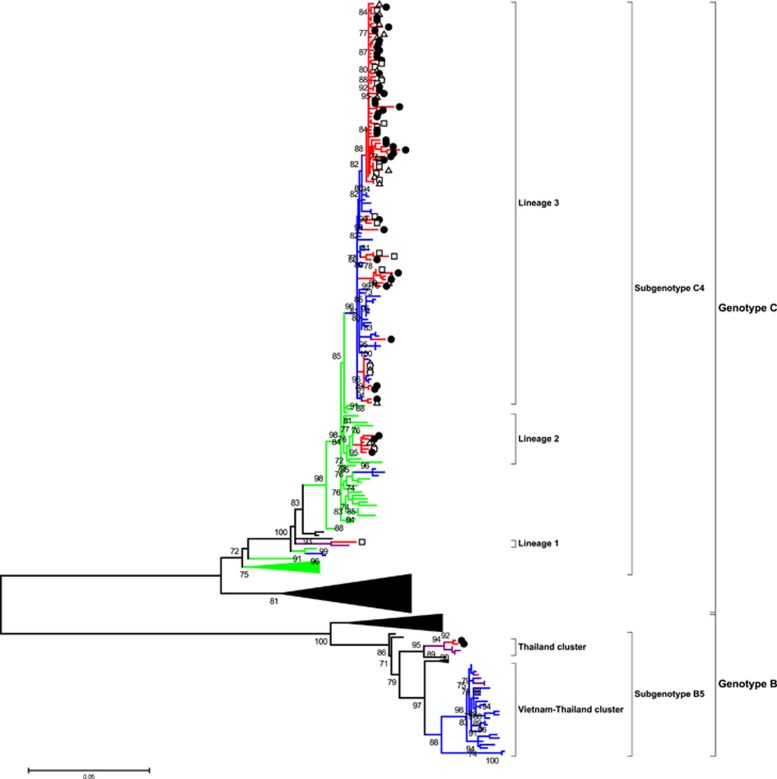
Maximum likelihood phylogenetic tree of 269 EV-A71 VP1 sequences. The branches are color-coded according to the location of sample collection (Cambodia=red, Vietnam=blue, Thailand=purple and China=green). For better clarity, taxon names are not shown, and sequences from other locations beside the four countries are not color-coded. The sequences isolated from Cambodian patients with different disease categories of severity are marked by white triangles for hand, foot and mouth disease, white rectangles for central nervous system involvement and black circles for cardiopulmonary failure. The tree was built using the maximum likelihood method based on the GTR+G4 model. The robustness of nodes was assessed with 1000 bootstrap replicates. Bootstrap values <70 are not shown.

The phylogenetic analysis of the VP1 gene sequence of EV-A71 viruses was based on 80 sequences from Cambodia. This included nine VP1 sequences in addition to 11 VP1 sequences that were from fully sequenced strains and that were generated in the present study by either Sanger or high-throughput sequencing sequencing methods (Table 1 and Supplementary Table S1). Additionally, 60 VP1 sequences were from full genomes of Cambodian strains generated in collaboration with the J. Craig Venter Institute and already available in GenBank. Phylogenetic analysis showed that all the strains that were isolated in Cambodia belonged to the C4a subgenotype except for three strains that clustered closely with the B5 subgenotype (Figure 1). A phylogenetic analysis of 70 full genomes of Cambodian EV-A71 strains was also conducted (Supplementary Figure S2). However, it did not demonstrate any significant difference compared with the VP1 gene analysis except for minor position changes, all within the same clusters. Consequently, only VP1 phylogeny will be discussed in this article. All the 77 Cambodian C4a strains clustered in three different lineages. Lineage 1 consisted of one strain that was detected in 2012 in Banteay Meanchey Province (Northwest region, close to the border with Thailand) that clustered closely to a Thai strain that was isolated in 2008. Five strains in lineage 2 were detected between 2012 and 2013 and were closely related to viruses originating from China between 2010 and 2012. Lineage 3 consisted of 76 Cambodian strains that were detected between 2012 and 2013 from different provinces across Cambodia that clustered with viruses that were isolated in Vietnam in 2011 and 2012. The three Cambodian subgenotype B5 strains were detected in 2012 in Banteay Meanchey Province (northwest region, close to the border with Thailand). Of the three B5 strains, two clustered with strains that were isolated in Thailand in 2011. One strain clustered with both Vietnamese strains that were isolated during the 2011–2013 epidemics as well as with Thai strains that were detected in 2012 (Figure 1).

We did not observe any association between the precise phylogeny of the Cambodian strains and the severity of disease or the geographical distribution of cases (data not shown).

There was 97.8% nucleotide identity between the VP1 and/or full-genome sequences of the Cambodian strains of lineage 1 and Thai strain FJ556875 THA08. Nucleotide identity of lineage 2 and the sequences of Chinese viruses isolated from 2010 to 2012 ranged from 97.4% to 99.1%. Nucleotide identity of lineage 3 and the sequences of the Vietnamese viruses that were isolated in 2011 ranged from 97.4% to 99.1%. The percentage of homology between the sequences of the Cambodian B5 subgenotype strains and the corresponding strains of Vietnamese and Thai origin with which they clustered ranged from 98.7% to 99.6% and from 98.7% to 99.0%, respectively.

**Figure 2:**
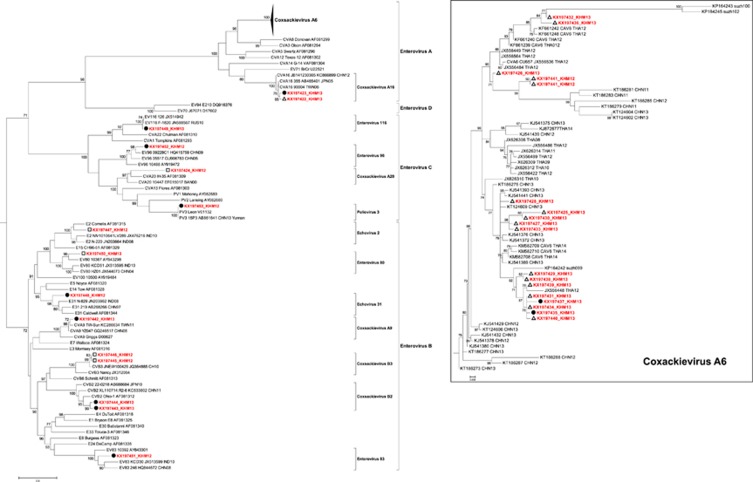
Maximum likelihood (ML) phylogenetic tree of 140 VP1 sequences from all four enterovirus species (A, B, C and D). The tree was built using the ML method based on the GTR+G4 model. The robustness of nodes was assessed with 1000 bootstrap replicates. Bootstrap values <70 are not shown. The sequences isolated from Cambodian patients with different disease categories of severity are marked by white triangles for hand, foot and mouth disease, white rectangles for central nervous system involvement and black circles for cardiopulmonary failure.

Phylogenetic analysis of 32 non-EV-A71 strains demonstrated that the Cambodian sequences belonged to three EV species (Figure 2), including 19 strains belonging to EV-A (17 Coxsackievirus (CV) A6 and 2 CV-A16), nine strains belonging to EV-B (1 EV-83, 2 CV-B3, 2 CV-B2, 1 CV-A9, 1 Echovirus (E)-31, 1 E-2 and 1 EV-B80) and four strains belonging to EV-C (one each of EV-C116, EV-C96, CV-A20 and poliovirus 3). The poliovirus 3 strain had a homology of >99% with a homologous Sabin vaccine strain and was classified as poliovirus vaccine-related. Of all non-EV-A71 viruses, CV-A6 was detected significantly more frequently in mild HFMD cases than in severe cases (*P*<0.001), while EV-B and EV-C were only found in the most severe cases (*P*<0.001; Table 1). Phylogenetic analysis of the CV-A6 sequences displayed close genetic relations to CV-A6 strains that circulated in Thailand and China (Figure 2).

### Mutations and recombination

We did not observe any of the known mutations that are associated with a specific clinical group. A recombination event was detected by five methods (RDP, Genecov, MaxChi, Chimaera and SiScan) in the RDP software with *P*-value <0.001 in one Cambodian EV-A71 strain (KP308450) collected in 2012. The recombination analysis detected two breakpoints at nucleotide positions 390 and 3664. The major parent was HM622391 (detected in Taiwan in 2008) and the minor parent was EF373576 (Taiwan, 2004) from the same genotype. No other recombination events were detected in the 2012–2013 Cambodian strains.

## DISCUSSION

This work describes the molecular characteristics of the EV-A71 viruses causing the first reported outbreak of EV infection with a high apparent number of severe outcomes in Cambodia between 2012 and 2013. The outbreak of HFMD in early July 2012 was marked by a very high CFR (92%) among patients at the intensive care units of Kantha Bopha Children's Hospitals. This caused panic among the public, which was abundantly reported by international media and commented in the scientific community.^25, 46, 47^ This study demonstrates that EV-A71 was the main etiological agent of the Cambodian outbreak. The circulation of various non-EV-A71 EV types was also detected. However, EVs in general are extremely common, especially in children, and cause a spectrum of usually mild infectious diseases that are rarely associated with epidemics. Their detection, essentially by rectal swabs, was therefore most likely not directly related to the outbreak of severe disease. Most patients originated from the Northeastern and Southern regions of Cambodia in which the two large pediatric hospitals participating in the study were located. These regions may represent a bias and limitation of geographical recruitment, especially as these hospitals provide care free of charge. Similar to other reports, young children were the most affected age group in our study with a median age of two years.^20, 48, 49^ We also observed that the proportion of girls was significantly higher in the clinically severe group. In a meta-analysis of 19 separate studies, however, gender was not a risk factor, whereas young age was a risk factor.^50^ Indeed, children aged below two years were often found to be at a higher risk of developing severe complications.^51, 52, 53^ We did not observe any difference in median age between the three clinical groups in the present study. The association of other factors with poor outcomes will be assessed in another article.

The phylogenetic analysis of 80 Cambodian strains demonstrated that the viruses clustered in subgenotypes C4a and B5. Subgenotype C4a has been reported to cause large outbreaks in Asia, including in China in 2012,^54^ Vietnam in 2011^28^ and Thailand in 2008–2009.^48^ Other outbreaks were reported with co-circulation of different genotypes or subgenotypes and other EVs of species such as in Vietnam in 2011 with subgenotypes EV-A71 C4 and C5,^28^ Thailand with EV-A71, CV-A16 and CV-A10^48^ and Singapore in which EV-A71 co-circulated with CV-A6, CV-A10, CV-A16 and CV-A4.^49^ Although subgenotype B5 was detected in Cambodia in a province bordering Thailand in 2012, this genotype was not detected subsequently. Genotype B5 virus was possibly introduced into Cambodia from neighboring Thailand or Vietnam where this virus circulated previously and caused outbreaks of HFMD in 2012^11, 12, 55^ if not earlier, according to serological studies conducted in Thailand.^12^ In 2012, Vietnam experienced a genotype switch from subgenogroup C4 to B5. Although the switch to B5 was associated with small outbreaks in Vietnam as in Sarawak (Malaysia) in 2003 and 2006,^56^ Taiwan experienced a large outbreak of subgenotype B5 strains in 2008.^57^

Interestingly, three distinct lineages were identified in subgenotype C4. Lineage 1 grouped closely to a Thai strain, lineage 2 was closely related to Chinese strains and lineage 3 clustered with Vietnamese strains. Cambodian B5 strains clustered with strains that were detected previously or concurrently in Thailand and Vietnam. This observation suggests several separate introductions of EV-A71 viruses from neighboring countries into Cambodia, in view of the very high percentage of nucleotide identity (>97%) between the Cambodian strains and those isolated in China, Vietnam and Thailand. The circulation of viruses between neighboring countries is often observed with EVs and has been regularly documented in the past.^58, 59, 60, 61^ Several genotypes (e.g., C1, C4, C5, B5) of EV-A71 and some non-EV-A71 viruses (e.g., CV-A16) circulated in Vietnam since 2005 and in Thailand since 2008,^12, 48^ with C5 being the predominant EV-A71 subgenotype.^20, 28^ The reason why HFMD was never detected or was detected but never reported in Cambodia before 2012 remains unclear. One of the best hypotheses is that the disease was misdiagnosed by clinicians. Indeed, our recent retrospective serological study using a EV-A71 strain isolated during the 2012 Cambodian outbreak to detect specific neutralizing antibodies showed that EV-A71 was widely circulating in the country at least during the preceding decade, with peaks in 2001, 2005, 2007 and 2010.^62^ These peaks coincided with outbreaks that occurred in Vietnam in 2005^28^ and 2011^20^ as well as in China and Taiwan in 2004–2005, 2008 and 2010–2012.^14, 53, 54, 63^

During the 2012 outbreak, many severe cases experiencing CPF did not present the typical rash usually observed in classic HFMD. Indeed, a papulovesicular rash was observed only in 14.7% of the CPF cases vs 72% in mild HFMD cases. Additionally, most admitted patients were critical and died within a few hours after admission. This patient outcome did not provide enough time for clinicians to conduct extensive diagnostic tests. The high CFR (92%) drew the attention of clinicians while many mild HFMD cases likely went unnoticed during the first outbreak period.

The high CFR was observed at the beginning of the outbreak in 2012 and then decreased slightly to an average of 63.3% among the patients presenting with CPF. The CFR in Cambodia was extremely high compared with that observed in other countries such as in China, Taiwan and Vietnam, where the CFR ranged from 0.14% to 34% depending on year, age group and gender.^52, 64, 65^ Subgenotype C4a, which was detected in most Cambodian cases, was already suspected to both cause more severe disease (CPF) and be associated with higher lethality.^50, 52, 66, 67, 68^ We did not find any significant molecular difference, however, between EV-A71 subgenotype C4a sequences isolated from mild HFMD and severe (CNSI and CPF) cases. Similar observations were also reported by other authors.^52, 69^ There may be other potential explanations for the severity of EV-A71 infections reported in Cambodia such as malnutrition, vitamin deficits, co-infection with other infectious diseases or delay in hospital admission combined with a lack of specific intensive care equipment (such as extracorporeal membrane oxygenation) or intravenous immunoglobulins.^50, 70, 71, 72, 73^ Host genetics may also have a role. Data to explore these hypotheses were not collected during the outbreak in 2012 and 2013. Further studies to explore the possible role of these factors would facilitate understanding the unique situation that Cambodia experienced in 2012. Co-infections involving several EVs were quite common in HFMD as well as in severe EV-A71 infections.^71, 74^ Four out of the five co-infections detected in this study, including the poliovirus vaccine-related EV, were associated with severe disease.

Recombinations are relatively common in EVs.^55, 75, 76^ A single case of recombination was detected in Cambodia; interestingly, the parent strain appeared to originate from Taiwan. Studies from Vietnam^55^ and Thailand^77^ also reported a low number of recombination events in their EV-A71 strains.

In addition to EV-A71, several other EVs were detected in the various clinical forms of the disease, including CV-A6, which was the most common of these non-EV-A71 viruses. In our study, CV-A6 was only associated with mild disease, while EV-A71 was detected in similar proportions among the three clinical forms. Our CV-A6 findings are consistent with other reports from the region.^49, 78, 79, 80, 81^ CV-A6 progressively became the most common virus detected among mild HFMD cases, apparently replacing other non-EV-A71 viruses, including CV-A16 in China, Thailand, Malaysia and Singapore.^11, 49, 82, 83, 84, 85, 86, 87^ CV-A6 seems now to be associated with many outbreaks worldwide.^88^ Unfortunately, no recent data regarding the circulation of CV-A6 in Vietnam is available. EV-B and EV-C species were only detected in severe (CNSI and CPF) cases. We cannot, however, confirm the association between these species and disease severity because none of these viruses were detected in CSF except for one case with CV-B3. In all other cases, the viruses were detected in other clinical specimens, especially in rectal swabs (17/32).

In this study, we report the identification of the origin of a large outbreak of EV-A71 and particularly of severe forms of central nervous system infections associated with fatal CPF. The main causative agent was EV-A71 genotype C4. The role of non-EV-A71 EVs such as CV-A6 in HFMD outbreaks will be studied further for potential consideration as a component of new vaccines to prevent HFMD. Physician training, improved reporting from Cambodian hospitals and cross-border surveillance should be implemented to deliver timely adequate care and curb the outbreak. Further study of the risk factors for severe HFMD, including host genetics, nutritional status, co-infection and comorbidity, should be investigated in order to better understand the pathogenesis of this disease.

## Figures and Tables

**Table 1:** Patient characteristics and symptoms/clinical signs

	**Total (*****n*****=312)**	**HFMD (*****n*****=142)**	**Central nervous system involvement (*****n*****=20)**	**Cardiopulmonary failure (*****n*****=150)**
*Age (years)*				
Mean (95% CI)	2.51	2.18^a^ (1.78–2.58)	2.51 (1.04–4.03)	2.84^b^ (2.28–3.40)
Median	2	2	1.7	2
Min.–max.	0.16–27	0.75–27	0.91–14	0.16–27
				
Sex (% female)	134 (42.8%)	52 (36.6%)	8 (40.0%)	74^c^ (50.3%)
				
*Hospitalization*				
No^d^	62 (20.0%)	62 (43.4%)	0	0
Hospitalized	250 (80.0%)	80 (56.6%)	20 (100%)	150 (100%)
				
*Clinical symptoms*				
Papulovesicular rash	130 (42.4%)	103 (72.0%)	5 (35.7%)	22 (14.7%)
Fatal cases	98 (31.4%)	0	3 (15.0%)	95 (63.3%)
				
*Laboratory diagnostic tests (RT-PCR and/or virus isolation)*				
EV-A71	208 (66.6%)	96 (67.6%)	11 (55.0%)	101 (67.3%)
Other than EV-A71	32 (10.3%)	18 (12.7%)	5 (25.0%)	9 (6.0%)
Subtyped	27 (73%)	15	5	7
Unsubtypable	5 (13.5%)	3	0	2
Co-infection with EV-A71 and non-EV-A71	5 (1.6%)	1 (0.7%)	0	4 (2.7%)
Negative	67 (21.5%)	27 (19.0%)	4 (20.0%)	36 (24.0%)
				
*Sequencing*				
EV-A71				
VP1	9	3 (33.3%)	4 (44.5%)	2 (22.2%)
Full genome	11	4 (36.5%)	2 (18.0%)	5 (45.5%)
Other than EV-A71				
VP1	32	16 (50.0%)	5 (15.6%)	11 (35.4%)
Enterovirus A	18 (56.3%)	14 CV-A6, 1 CV-A16		2 CV-A6, 1 CV-A16
Enterovirus B	7 (21.9%)	0	2 CV-B3, 1 E-2 and 1 EV-B80	1 EV-B83, 1 CV-B2, 1 E-31 and 1 CV-A9
Enterovirus C	2 (6.3%)	0	1 CV-A20	1 EV-C116
Co-infection with EV-A71	5 (15.5%)	1 CV-A6 (A)	0	2 CV-B2 (B), 1 EV-C96 (C) and 1 PV-3 (C)

Abbreviations: enterovirus A, (A); enterovirus B, (B); enterovirus C, (C); confidence interval, CI; coxsackievirus A, CVA; coxsackievirus B, CVB; echovirus, E; enterovirus, EV; human enterovirus 71, EV-A71; hand, foot and mouth disease, HFMD; poliovirus, P; reverse transcription PCR, RT-PCR; poliovirus E vaccine-related, VRPV.

a *n*=141.

b *n*=146.

c *n*=147.

d Patients recruited at the outpatient department.
