# Supplementary material for: Molecular epidemiology of human enterovirus 71 at the origin of an epidemic of fatal hand, foot and mouth disease cases in Cambodia
Source: Emerg Microbes Infect. 2016 Sep 21;5(9):e104–. doi: 10.1038/emi.2016.101 (PMC5113052; doi:10.1038/emi.2016.101)
Supplement: Supplementary Table S1 [file emi2016101x1.doc]

|  | Primer’s name | Primer’s sequence (5’ – 3’) |
| --- | --- | --- |
| **Fragment 1** | FL-F | TTAAAACAGCCTGTGGGTTGCACCCACTC |
|  | 556R | GACACCCAAAGTAGTCGGTTCCG |
| Fragment 2 | | 447F | | --- | | | GTCCTCCGGCCCCTGAATGCG | | --- | |
|  | 1075R | CAGTAKGARGGCCACTCMCC |
| **Fragment 3** | 863S | GGGCAAACAGAGTCTCAAGC |
|  | 1948A | GCGCAGTCTCTCCATTAAGC |
| **Fragment 4** | 1767S | GTTTCGGCACCTATTCTACC |
|  | 2578A | TGGAGTGCTGGAACCTTACC |
| **Fragment 5** | 2372S(VP1) | GCAGCCCAAAAGAACTTCAC |
|  | 3454A(VP1) | AAGTCGCGAGAGCTGTCTTC |
| **Fragment 6** | 3192F | TAYATGAGRATGAARCAYGTCAG |
|  | 4430R | GTGYKTGCTCTTGAACTGCAT |
| **Fragment 7** | 4181S | AGTACCAGCAGCCAAGGAGA |
|  | 5207A | GGTGGGAGTTTCAGGAATGA |
| **Fragment 8** | 5068S | CACAATCGAGGCTCTTTTCC |
|  | 6195A | GCTGCCTCTTTGATGTACTCG |
| **Fragment 9** | 5897S | CGCAGGCCTTAAAAGGAGTT |
|  | 6749A | TGTGTGGTTGATTCCCTCAA |
| **Fragment 10** | 6602S | TTTGCTCCCTGGTTCACTCT |
|  | FL-R (cDNA primer) | TTTTTTTTTTGCTATTCYGGTTATAACAAAT |

Supplementary Table S1 Sequence of the primers used in the study
