# Supplementary material for: Molecular epidemiology of human enterovirus 71 at the origin of an epidemic of fatal hand, foot and mouth disease cases in Cambodia
Source: Emerg Microbes Infect. 2016 Sep 21;5(9):e104–. doi: 10.1038/emi.2016.101 (PMC5113052; doi:10.1038/emi.2016.101)
Supplement: Supplementary Table S2 [file emi2016101x2.doc]

Suplementary Table S2 Detailed information on patients, diagnostic and sequencing results

| **Patient's ID** | **Age (year)** | **Sex** | **Province** | **Clinical classification** | **Date of sampling** | **Type of clinical specimen positive by PCR and/or culture** | **Enterovirus classification** | **Sequence** | **Sequencing technique** | **Sequencing facility** | **Strain** | **GenBank accession number** |
| --- | --- | --- | --- | --- | --- | --- | --- | --- | --- | --- | --- | --- |
| HFM002 | 2 | Female | Phnom Penh | CNSI | 26-Jun-12 | Throat & rectal* swabs | EV-A71 | VP1 | Sanger | IP Cambodia | In this study | KX197414 |
| HFM022 | 2 | Female | Battambang | CNSI | 12-Jul-12 | Throat & rectal* swabs | EV-A71 | VP1 | Sanger | IP Cambodia | In this study | KX197415 |
| HFM025 | 3 | Male | Banteay Meanchey | CNSI | 13-Jul-12 | Throat & rectal* swabs | EV-A71 | VP1 | Sanger | IP Cambodia | In this study | KX197416 |
| HFM026 | 1 | Female | Phnom Penh | CNSI | 13-Jul-12 | Throat & rectal* swabs | EV-A71 | VP1 | Sanger | IP Cambodia | In this study | KX197417 |
| HFM030 | 3 | Male | Phnom Penh | HFMD | 13-Jul-12 | Throat & rectal* swabs | EV-A71 | VP1 | Sanger | IP Cambodia | In this study | KX197418 |
| HFM031 | 2 | Male | Kampong Cham | HFMD | 13-Jul-12 | Throat & rectal* swabs | EV-A71 | VP1 | Sanger | IP Cambodia | In this study | KX197419 |
| HFM205 | 2 | Male | Kampong Speu | HFMD | 23-Jan-13 | Serum, throat & rectal* swabs | EV-A71 | VP1 | Sanger | IP Cambodia | In this study | KX197420 |
| SEP040 | 0.25 | Male | Siem reap | CPF | 11-Jul-12 | Serum* & rectal swab / BAL*, throat & rectal swabs | EV-A71/  PV-3 | VP1/VP1 | Sanger | IP Cambodia | In this study | KX197421/  KX197453 |
| SEP143 | 3 | Female | Kandal | CPF | 9-Jul-13 | Throat* & rectal swabs | EV-A71 | VP1 | Sanger | IP Cambodia | In this study | KX197422 |
| HFM005 | 2 | Female | Kampong Speu | CNSI | 8-Jul-12 | Throat* & rectal swabs | EV-A71 | Complete genome | Sanger | IP Cambodia | In this study | KX197455 |
| HFM008 | 5 | Male | Kampong Cham | HFMD | 12-Jul-12 | Throat* & rectal swabs | EV-A71 | Complete genome | Sanger | IP Cambodia | In this study | KX197456 |
| HFM040 | 4 | Female | Kampong Chnang | HFMD | 14-Jul-12 | Throat* & rectal swabs | EV-A71 | Complete genome | HTS | IP Paris | In this study | KX197457 |
| HFM057 | 2 | Female | Banteay Meanchey | CNSI | 14-Jul-12 | Serum, throat & rectal* swabs | EV-A71 | Complete genome | Sanger | IP Cambodia | In this study | KX197458 |
| HFM072 | 2 | Male | Pailin | HFMD | 15-Jul-12 | BAL, throat & rectal* swabs | EV-A71 | Complete genome | HTS | IP Shanghai | In this study | KX197459 |
| HFM088 | 1 | Male | Phnom Penh | HFMD | 17-Jul-12 | Throat* & rectal swabs | EV-A71 | Complete genome | HTS | IP Paris | In this study | KX197460 |
| SEP001 | 3 | Male | NA | CPF | 15-Jun-12 | CSF, throat* & rectal swabs | EV-A71 | Complete genome | HTS | HKU | In this study | KX197461 |
| SEP006 | 0.75 | Female | Pursat | CPF | 23-Jun-12 | Throat* & rectal swabs | EV-A71 | Complete genome | HTS | IP Paris | In this study | KX197462 |
| SEP014 | 1 | Male | Kampong Cham | CPF | 28-Jun-12 | CSF*, throat & rectal swabs / Throat* swab | EV-A71/  EV-C96 | Complete genome/VP1 | HTS | IP Paris | In this study | KX197463/  KX197452 |
| SEP024 | 2 | Female | Takeo | CPF | 7-Jul-12 | Throat* & rectal swabs | EV-A71 | Complete genome | HTS | IP Paris | In this study | KX197464 |
| SEP042 | 2 | Male | Battambang | CPF | 2012 | Serum, throat* & rectal swabs | EV-A71 | Complete genome | Sanger | IP Cambodia | In this study | KX197465 |
| HFM062 | 1 | Male | Battambang | HFMD | 14-Jul-12 | Throat* & rectal swabs | CV-A6 | VP1 | Sanger | IP Cambodia | In this study | KX197441 |
| HFM178 | 3 | Male | Kampong Cham | HFMD | 2013 | Rectal* swab | CV-A6 | VP1 | Sanger | IP Cambodia | In this study | KX197425 |
| HFM179 | 3 | Female | Prey Veng | HFMD | 2013 | Throat* swab | CV-A16 | VP1 | Sanger | IP Cambodia | In this study | KX197422 |
| HFM181 | 1 | Male | Kandal | HFMD | 2013 | Rectal* swab | CV-A6 | VP1 | Sanger | IP Cambodia | In this study | KX197426 |
| HFM182 | 1 | Female | Kandal | HFMD | 2013 | Throat & rectal* swabs | CV-A6 | VP1 | Sanger | IP Cambodia | In this study | KX197427 |
| HFM183 | 6 | Male | Phnom Penh | HFMD | 2013 | Throat & rectal* swabs | CV-A6 | VP1 | Sanger | IP Cambodia | In this study | KX197428 |
| HFM185 | 0.75 | Female | Kampong Cham | HFMD | 2013 | Throat* & rectal swabs | CV-A6 | VP1 | Sanger | IP Cambodia | In this study | KX197429 |
| HFM186 | 1 | Male | Kampong Cham | HFMD | 2013 | Throat & rectal* swabs | CV-A6 | VP1 | Sanger | IP Cambodia | In this study | KX197430 |
| HFM188 | 1 | Female | Phnom Penh | HFMD | 2013 | Rectal* swab | CV-A6 | VP1 | Sanger | IP Cambodia | In this study | KX197431 |
| HFM189 | 1 | Male | Kampong Cham | HFMD | 11-Jan-13 | Rectal* swab | CV-A6 | VP1 | Sanger | IP Cambodia | In this study | KX197432 |
| HFM191 | 3 | Male | Phnom Penh | HFMD | 11-Jan-13 | Rectal* swab | CV-A6 | VP1 | Sanger | IP Cambodia | In this study | KX197433 |
| HFM192 | 1 | Male | Kandal | HFMD | 16-Jan-13 | Throat & rectal* swabs | CV-A6 | VP1 | Sanger | IP Cambodia | In this study | KX197434 |
| HFM197 | 2 | Male | Kampong Cham | HFMD | 18-Jan-13 | Rectal* swab | CV-A6 | VP1 | Sanger | IP Cambodia | In this study | KX197436 |
| HFM203 | 1 | Female | Kandal | HFMD | 22-Jan-13 | Rectal swab/ Throat* swab | EV-A71/  CV-A6 | NA/VP1 | Sanger | IP Cambodia | In this study | KX197438 |
| HFM206 | 1 | Male | Phnom Penh | HFMD | 2013 | Throat & rectal* swabs | CV-A6 | VP1 | Sanger | IP Cambodia | In this study | KX197439 |
| HFM210 | 1 | Male | Prey Veng | HFMD | 31-Jan-13 | Throat* & rectal swabs | CV-A6 | VP1 | Sanger | IP Cambodia | In this study | KX197440 |
| SEP193 | NA | Female | Prey Veng | CPF | 16-Jan-13 | Serum*, throat & rectal swabs | CV-A6 | VP1 | Sanger | IP Cambodia | In this study | KX197435 |
| SEP198 | 6 | Male | Prey Veng | CPF | 18-Jan-13 | Rectal* swab | CV-A6 | VP1 | Sanger | IP Cambodia | In this study | KX197437 |
| ME046 | 1.6 | Female | Siem Reap | CNSI | 11-Sep-10 | Rectal* swab | CV-A20 | VP1 | Sanger | IP Cambodia | In this study | KX197424 |
| ME298 | 1.8 | Female | Siem Reap | CNSI | 19-Jan-12 | Rectal* swab | E-2 | VP1 | Sanger | IP Cambodia | In this study | KX197447 |
| ME313 | 1.8 | Male | Kampong Thom | CNSI | 3-Feb-12 | Throat & rectal* swabs | CV-B3 | VP1 | Sanger | IP Cambodia | In this study | KX197445 |
| ME316 | 1.6 | Female | Kampong Thom | CNSI | 10-Feb-12 | CSF* | CV-B3 | VP1 | Sanger | IP Cambodia | In this study | KX197446 |
| ME1161 | 3 | Male | Prey Veng | CNSI | 01-Oct-13 | Rectal* swab | EV-B80 | VP1 | Sanger | IP Cambodia | In this study | KX197450 |
| SEP025 | 4.75 | Female | Kampong Chhnang | CPF | 5-Jul-12 | Rectal* swab | EV-B83 | VP1 | Sanger | IP Cambodia | In this study | KX197451 |
| SEP129 | 2 | Female | Battambang | CPF | 14-Mar-13 | BAL*, throat & rectal* swabs | CV-A9 | VP1 | Sanger | IP Cambodia | In this study | KX197442 |
| SEP134 | 1 | Female | Kampong Cham | CPF | 17-Apr-13 | Rectal* swab | E-31 | VP1 | Sanger | IP Cambodia | In this study | KX197448 |
| SEP136 | 3 | Male | Kandal | CPF | 12-May-13 | Rectal* swab | E-116 | VP1 | Sanger | IP Cambodia | In this study | KX197449 |
| SEP146 | 2 | Female | Prey Veng | CPF | 7-Sep-13 | Serum, CSF throat* & rectal swabs/Throat* swab | EV-A71/  CV-B2 | NA/VP1 | Sanger | IP Cambodia | In this study | KX197443 |
| SEP148 | 1 | Female | Phnom Penh | CPF | 20-Sep-13 | Throat & rectal* swabs | CV-A16 | VP1 | Sanger | IP Cambodia | In this study | KX197423 |
| SEP151 | 1 | Female | Kampong Cham | CPF | 12-Oct-13 | Throat & rectal swabs/Rectal* swab | EV-A71/  CV-B2 | NA/VP1 | Sanger | IP Cambodia | In this study | KX197444 |
| SEP004 | 0.5 | Female | Kampong Cham | CPF | 15-Jun-12 | Serum, CSF throat & rectal* swabs | EV-A71 | Complete genome | HTS | HKU & IP Paris | GenBank | KF543271 |
| SEP031 | 15 | Female | NA | CPF | 9-Jul-12 | Rectal* swab | EV-A71 | Complete genome | HTS | NIAID | GenBank | KP308401 |
| SEP115 | 1.4 | Female | Kampong Thom | CPF | 13-Nov-12 | Throat* & rectal swabs | EV-A71 | Complete genome | HTS | NIAID | GenBank | KP308402 |
| SEP085 | 0.5 | Female | Prey Veng | CPF | 26-Aug-12 | CSF throat* & rectal swabs | EV-A71 | Complete genome | HTS | NIAID | GenBank | KP308403 |
| SEP114 | 0.11 | Male | NA | CPF | 7-Nov-12 | CSF throat* & rectal swabs | EV-A71 | Complete genome | HTS | NIAID | GenBank | KP308404 |
| HFM105 | 1 | Male | NA | CNSI | 16-Jul-12 | Throat & rectal* swabs | EV-A71 | Complete genome | HTS | NIAID | GenBank | KP308405 |
| HFM171 | 9.3 | Male | Banteay Meanchey | CPF | 31-Aug-12 | Throat* & rectal swabs | EV-A71 | Complete genome | HTS | NIAID | GenBank | KP308406 |
| HFM120 | 0.3 | Male | Kampong Speu | CPF | 17-Jul-12 | Throat* & rectal swabs | EV-A71 | Complete genome | HTS | NIAID | GenBank | KP308407 |
| SEP102 | 2.5 | Male | Battambang | CPF | 16-Sep-12 | Throat* & rectal swabs | EV-A71 | Complete genome | HTS | NIAID | GenBank | KP308408 |
| HFM044 | 1.2 | Female | Prey Veng | CNSI | 14-Jul-12 | Serum, throat* & rectal swabs | EV-A71 | Complete genome | HTS | NIAID | GenBank | KP308409 |
| HFM025 | 2.4 | Male | Banteay Meanchey | CNSI | 13-Jul-12 | Throat & rectal* swabs | EV-A71 | Complete genome | HTS | NIAID | GenBank | KP308410 |
| HFM042 | 2.7 | Female | Kandal | HFMD | 14-Jul-12 | Throat & rectal* swabs | EV-A71 | Complete genome | HTS | NIAID | GenBank | KP308411 |
| HFM106 | 1 | Female | Siem Reap | CNSI | 16-Jul-12 | Throat & rectal* swabs | EV-A71 | Complete genome | HTS | NIAID | GenBank | KP308412 |
| HFM205 | 2 | Male | Kampong Speu | HFMD | 23-Jan-13 | Serum, throat & rectal* swabs | EV-A71 | Complete genome | HTS | NIAID | GenBank | KP308413 |
| SEP063 | 2 | Male | Kampong Cham | CPF | 30-Jul-12 | Throat* & rectal swabs | EV-A71 | Complete genome | HTS | NIAID | GenBank | KP308414 |
| SEP055 | 1.5 | Male | Siem Reap | CPF | 24-Jul-12 | CSF, BAL, throat & rectal* swabs | EV-A71 | Complete genome | HTS | NIAID | GenBank | KP308415 |
| HFM045 | 1.9 | Male | Kampong Chnang | CNSI | 14-Jul-12 | Throat & rectal* swabs | EV-A71 | Complete genome | HTS | NIAID | GenBank | KP308416 |
| SEP072 | 2 | Female | NA | CPF | 9-Aug-12 | CSF, throat & rectal* swabs | EV-A71 | Complete genome | HTS | NIAID | GenBank | KP308417 |
| HFM132 | 7.3 | Female | NA | NA | 21-Jul-12 | Throat* & rectal swabs | EV-A71 | Complete genome | HTS | NIAID | GenBank | KP308418 |
| HFM043 | 1.9 | Male | Phnom Penh | HFMD | 14-Jul-12 | Throat* & rectal swabs | EV-A71 | Complete genome | HTS | NIAID | GenBank | KP308419 |
| SEP071 | 2 | Male | Pailin | CPF | 9-Aug-12 | CSF, throat & rectal* swabs | EV-A71 | Complete genome | HTS | NIAID | GenBank | KP308420 |
| SEP088 | 1 | Male | NA | CPF | 25-Aug-12 | CSF, throat* & rectal swabs | EV-A71 | Complete genome | HTS | NIAID | GenBank | KP308421 |
| HFM055 | 1.3 | Male | Phnom Penh | HFMD | 16-Jul-12 | Throat* & rectal swabs | EV-A71 | Complete genome | HTS | NIAID | GenBank | KP308422 |
| HFM054 | 2 | Male | Prey Veng | HFMD | 16-Jul-12 | Throat* & rectal swabs | EV-A71 | Complete genome | HTS | NIAID | GenBank | KP308423 |
| HFM124 | 1.2 | Male | Takeo | CNSI | 19-Jul-12 | Throat* & rectal swabs | EV-A71 | Complete genome | HTS | NIAID | GenBank | KP308424 |
| HFM103 | 1 | Female | Kampong Speu | CNSI | 17-Jul-12 | Rectal* swab | EV-A71 | Complete genome | HTS | NIAID | GenBank | KP308425 |
| SEP149 | NA | Male | Kampong Cham | CPF | 23-Sep-13 | Serum and throat* swab | EV-A71 | Complete genome | HTS | NIAID | GenBank | KP308426 |
| SEP120 | 0.1 | Male | Banteay Meanchey | CPF | 6-Dec-12 | Serum, CSF, throat* & rectal swabs | EV-A71 | Complete genome | HTS | NIAID | GenBank | KP308427 |
| SEP074 | 1 | NA | Kampong Thom | CPF | 12-Aug-12 | CSF, BAL* & rectal swab | EV-A71 | Complete genome | HTS | NIAID | GenBank | KP308428 |
| HFM052 | 4 | Male | Kratie | HFMD | 16-Jul-12 | Throat* & rectal swabs | EV-A71 | Complete genome | HTS | NIAID | GenBank | KP308429 |
| SEP067 | 1 | Male | Banteay Meanchey | CPF | 7-Aug-12 | CSF, BAL, throat* & rectal swabs | EV-A71 | Complete genome | HTS | NIAID | GenBank | KP308430 |
| SEP124 | 1 | Male | Svay Reang | CPF | 6-Feb-13 | CSF, throat* & rectal swabs | EV-A71 | Complete genome | HTS | NIAID | GenBank | KP308431 |
| SEP058 | 1 | Male | Kampong Speu | CPF | 29-Jul-12 | Serum, CSF*, throat & rectal swabs | EV-A71 | Complete genome | HTS | NIAID | GenBank | KP308432 |
| SEP057 | 2 | Male | Kampong Cham | CPF | 29-Jul-12 | CSF, throat & rectal* swabs | EV-A71 | Complete genome | HTS | NIAID | GenBank | KP308433 |
| SEP087 | 1 | Male | Battambang | CPF | 25-Aug-12 | CSF, BAL*, throat & rectal swabs | EV-A71 | Complete genome | HTS | NIAID | GenBank | KP308434 |
| SEP121 | 2 | Male | Kampong Cham | CPF | 8-Dec-12 | Throat* swab | EV-A71 | Complete genome | HTS | NIAID | GenBank | KP308435 |
| SEP083 | 1 | Female | Battambang | CPF | 21-Aug-12 | BAL, throat* & rectal swabs | EV-A71 | Complete genome | HTS | NIAID | GenBank | KP308436 |
| SEP080 | 1 | Female | Banteay Meanchey | CPF | 20-Aug-12 | Throat* & rectal swabs | EV-A71 | Complete genome | HTS | NIAID | GenBank | KP308437 |
| SEP100 | 2 | Male | Kandal | CPF | 9-Sep-12 | CSF, throat & rectal* swabs | EV-A71 | Complete genome | HTS | NIAID | GenBank | KP308438 |
| SEP117 | 1.6 | Male | Kampong Thom | CPF | 23-Nov-12 | Serum, throat* & rectal swabs | EV-A71 | Complete genome | HTS | NIAID | GenBank | KP308439 |
| HFM104 | 2 | Female | Battambang | CNSI | 16-Jul-12 | Throat & rectal* swabs | EV-A71 | Complete genome | HTS | NIAID | GenBank | KP308440 |
| SEP061 | NA | NA | Battambang | CPF | 31-Jul-12 | BAL, throat & rectal* swabs | EV-A71 | Complete genome | HTS | NIAID | GenBank | KP308441 |
| HFM063 | 1.1 | Female | Siem Reap | HFMD | 14-Jul-12 | Throat* & rectal swabs | EV-A71 | Complete genome | HTS | NIAID | GenBank | KP308442 |
| HFM138 | 1.6 | Female | Kampot | CNSI | 25-Jul-12 | Throat* & rectal swabs | EV-A71 | Complete genome | HTS | NIAID | GenBank | KP308443 |
| HFM174 | 0.6 | Male | Kampong Thom | NA | 16-Jul-12 | Throat & rectal* swabs | EV-A71 | Complete genome | HTS | NIAID | GenBank | KP308444 |
| HFM012 | 1.2 | Male | Kandal | CNSI | 12-Jul-12 | Throat* & rectal swabs | EV-A71 | Complete genome | HTS | NIAID | GenBank | KP308445 |
| HFM053 | 2.9 | Male | Kampong Som | HFMD | 16-Jul-12 | Throat & rectal* swabs | EV-A71 | Complete genome | HTS | NIAID | GenBank | KP308446 |
| HFM036 | 1.2 | Female | Prey Veng | HFMD | 16-Jul-12 | Throat & rectal* swabs | EV-A71 | Complete genome | HTS | NIAID | GenBank | KP308447 |
| SEP060 | 1 | Male | Banteay Meanchey | CPF | 29-Jul-12 | Throat* & rectal swabs | EV-A71 | Complete genome | HTS | NIAID | GenBank | KP308448 |
| HFM107 | 1 | Female | Siem Reap | CNSI | 16-Jul-12 | Throat* & rectal swabs | EV-A71 | Complete genome | HTS | NIAID | GenBank | KP308449 |
| HFM061 | 1.5 | Female | Banteay Meanchey | CNSI | 14-Jul-12 | Throat* & rectal swabs | EV-A71 | Complete genome | HTS | NIAID | GenBank | KP308450 |
| SEP098 | 2 | Male | NA | CPF | 6-Sep-12 | Throat* & rectal swabs | EV-A71 | Complete genome | HTS | NIAID | GenBank | KP308451 |
| HFM136 | 2 | Male | Takeo | CNSI | 24-Jul-12 | Throat* & rectal swabs | EV-A71 | Complete genome | HTS | NIAID | GenBank | KP308452 |
| SEP051 | 3 | Male | Banteay Meanchey | CPF | 24-Jul-12 | Throat* & rectal swabs | EV-A71 | Complete genome | HTS | NIAID | GenBank | KP308453 |
| HFM070 | 5 | Male | Banteay Meanchey | NA | 14-Jul-12 | Throat* swab | EV-A71 | Complete genome | HTS | NIAID | GenBank | KP308454 |
| SEP113 | 1.6 | Female | Kampong Cham | CPF | 5-Nov-12 | Throat* & rectal swabs | EV-A71 | Complete genome | HTS | NIAID | GenBank | KP308456 |
| HFM114 | 1 | Male | Kampong Cham | HFMD | 17-Jun-12 | Throat & rectal* swabs | EV-A71 | Complete genome | HTS | NIAID | GenBank | KP308457 |
| SEP127 | 0.9 | Female | Kampot | CPF | 25-Feb-13 | BAL, throat & rectal* swabs | EV-A71 | Complete genome | HTS | NIAID | GenBank | KP308458 |
| HFM018 | 1 | Male | Banteay Meanchey | HFMD | 12-Jul-12 | Throat* & rectal swabs | EV-A71 | Complete genome | HTS | NIAID | GenBank | KP308459 |
| HFM208 | 2 | Male | Oudar Meanchey | CPF | 29-Jan-13 | Serum, throat* & rectal swabs | EV-A71 | Complete genome | HTS | NIAID | GenBank | KP308460 |

NIAID: National Institute of Allergy and Infectious Diseases

HKU: Hong Kong University

IP: Institut Pasteur

HTS: High Throughput Sequencing

HFMD: Hand, foot and mouth disease

CNSI: Central nervous system involvement

CPF: Cardiopulmonary failure

CV: Coxsackievirus

EV: Enterovirus

E: Echovirus

PV: Poliovirus

NA: Not avalable

BAL: Bronchoalveolar lavage

* Clinical specimen used to generate the sequences

CSF: Cerebrospinal fluid
