# Supplementary material for: Molecular epidemiology of human enterovirus 71 at the origin of an epidemic of fatal hand, foot and mouth disease cases in Cambodia
Source: Emerg Microbes Infect. 2016 Sep 21;5(9):e104–. doi: 10.1038/emi.2016.101 (PMC5113052; doi:10.1038/emi.2016.101)
Supplement: Supplementary Figure S1 [file emi2016101x3.pdf]

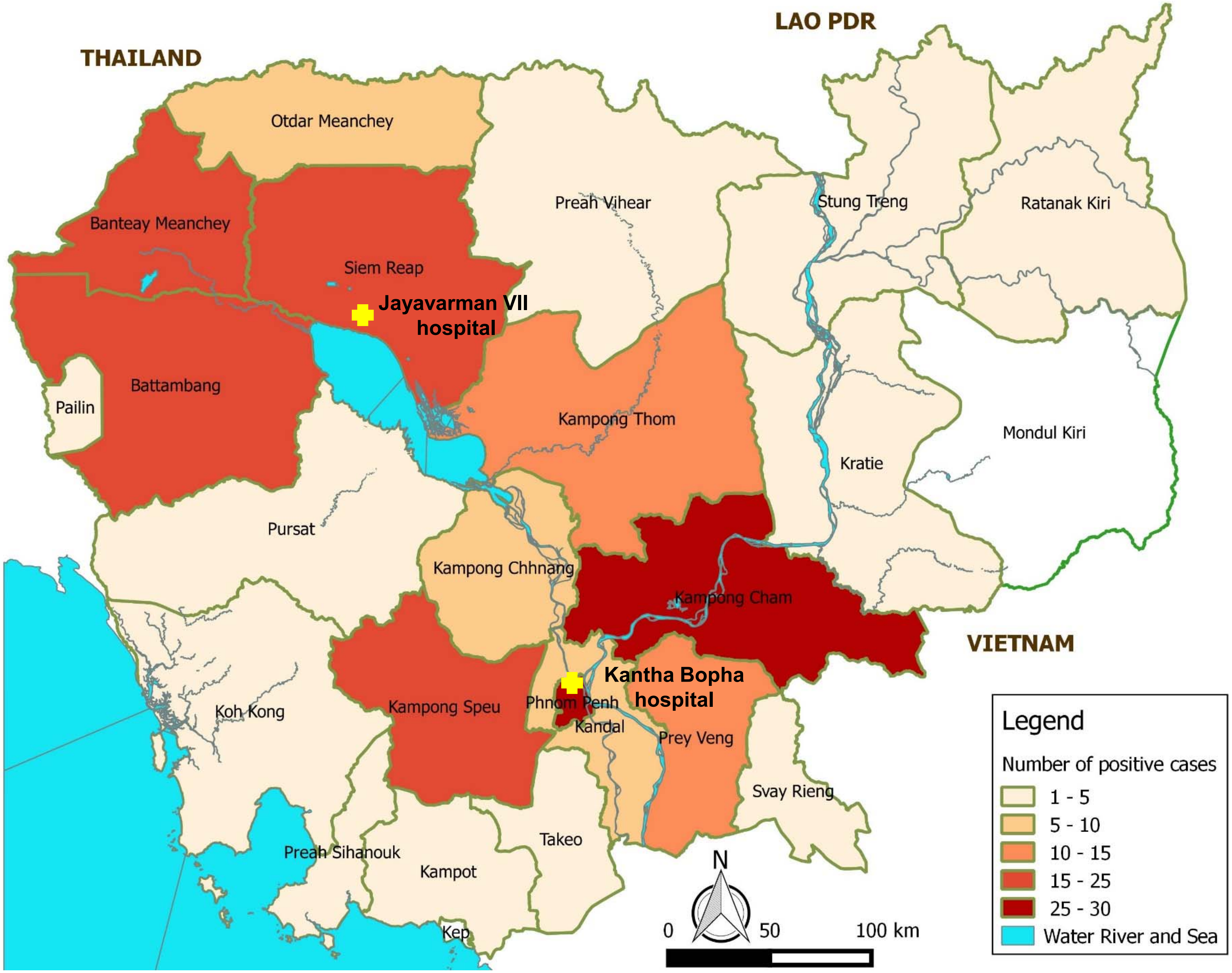

Supplementary Figure S1 Map of Cambodia showing the number of EV-A71 positive cases by province. White color means that no cases occurred.
