# Supplementary material for: Molecular epidemiology of human enterovirus 71 at the origin of an epidemic of fatal hand, foot and mouth disease cases in Cambodia
Source: Emerg Microbes Infect. 2016 Sep 21;5(9):e104–. doi: 10.1038/emi.2016.101 (PMC5113052; doi:10.1038/emi.2016.101)
Supplement: Supplementary Figure S2 [file emi2016101x4.pdf]

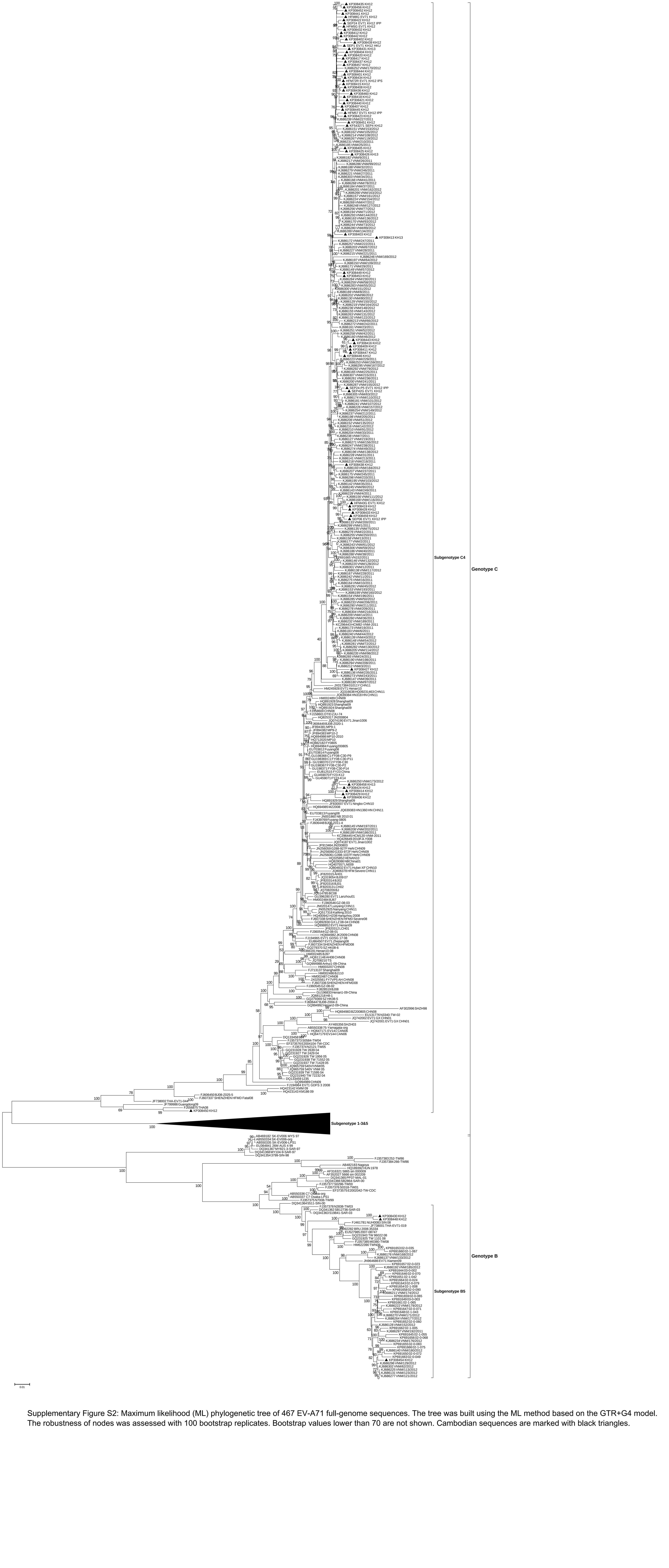

Supplementary Figure S2: Maximum likelihood (ML) phylogenetic tree of 467 EV-A71 full-genome sequences. The tree was built using the ML method based on the GTR+G4 model. The robustness of nodes was assessed with 100 bootstrap replicates. Bootstrap values lower than 70 are not shown. Cambodian sequences are marked with black triangles.
